# Wording the trajectory of the three-year COVID-19 epidemic in a general population – Belgium

**DOI:** 10.1186/s12889-024-17951-x

**Published:** 2024-02-29

**Authors:** Jean Vanderpas, Michèle Dramaix, Yves Coppieters

**Affiliations:** Centre de recherche Epidémiologie, biostatistiques, recherche clinique, School of Public Health, Université libre de Bruxelles (ULB), Route de Lennik 808, 596, 1070 Brussels, CP Belgium

**Keywords:** Infectious disease dynamic, SIR model

## Abstract

The trajectory of COVID-19 epidemic waves in the general population of Belgium was analysed by defining quantitative criteria for epidemic waves from March 2020 to early 2023. Peaks and starting/ending times characterised nine waves numerated I to IX based on the daily reported incidence number (symbol INCID) and three “endemic” interval periods between the first four waves. The SIR compartmental model was applied to the first epidemic wave by fitting the daily prevalence pool (symbol I) calculated as the sum of the daily incidence rate and estimated number of subjects still infectious from the previous days. The basic reproductive number R_0_ was calculated based on the exponential growth rate during the early phase and on medical literature knowledge of the time of generation of SARS-CoV-2 infection. The first COVID-19 wave was well fitted by an open SIR model. According to this approach, dampened recurrent epidemic waves evolving through an endemic state would have been expected. This was not the case with the subsequent epidemic waves being characterised by new variants of concern (VOC). Evidence-based observations: 1) each epidemic wave affected less than a fifth of the general population; 2) the Vth epidemic wave (VOC Omicron) presented the greatest amplitude. The lack of recurrence of the same VOC during successive epidemic waves strongly suggests that a VOC has a limited persistence, disappearing from the population well before the expected proportion of the theoretical susceptible cohort being maximally infected. Fitting the theoretical SIR model, a limited persistence of VOCs in a population could explain that new VOCs replace old ones, even if the new VOC has a lower transmission rate than the preceding one. In conclusion, acquisition of potential defective mutations in VOC during an epidemic wave is a potential factor explaining the absence of resurgence of a same VOC during successive waves. Such an hypothesis is open to discussion and to rebuttal. A modified SIR model with epidemic waves of variable amplitude related not only to R_0_ and public health measures but also to acquisition of defective fitting in virus within a population should be tested.

**Supplementary Information:**

The online version contains supplementary material available at 10.1186/s12889-024-17951-x.

## Introduction

Specific definitions describe the mode of propagation of a pathogen agent in a cohort or in a population. While these definitions look easy to understand intuitively, they are relatively difficult to convert into a unified method of measurement. In the original background publications of Ross and Kermack [1, 2], the mathematical development of infectious epidemiology prioritised the pragmatic usage of measurement tools. During the three-year COVID-19 pandemic, the same paradox was observed: on the one hand, mathematicians/bioinformatics analysts developed very refined models of dynamic infection by integrating various subgroups of a population according to age, sex, living at home or type of institution, travel, nonpharmaceutical and vaccination intervention [3]; on the other hand, health professionals were pragmatically involved in collecting basic data such as daily incidence rates of reported COVID-19 cases [4] and seroprevalence data of SARS-CoV-2 immunity, at least in the general population [5], as required by international survey organisations during a pandemic. Communication between both groups of professionals was limited, not only for different backgrounds of knowledge but also for difficulties in sharing the same scientific language. The manuscript goes back to some basic methodological questions on SIR model from experts in epidemiology, in continuation of the pre-Covid experience obtained in the field of infectious epidemiology diffused through courses at Imperial College, London (Fraser Christophe and colleagues, 2018) [6], the need of clarification being reinforced by the mass of literature since the emergence of Covid (more than 9 000 publications from 2020 to present with key words “Covid SIR model”).

### Do we agree on the definition of I in the SIR model ?

When discussing with health professionals in the field, a frequently erroneous understanding of “I” in the infectious dynamic SIR model refers intuitively and erroneously to “incidence rate” in place of “prevalence pool of infected/infectious cases” [7]. In addition to conceptual mistakes, such misunderstandings could have consequences on modelling the SIR/SEIR model and on the measurement of basic reproductive number R_0_. In the present pragmatic approach, it is proposed to show step by step how to integrate field observations by infection control teams with the SIR model in a general population. The classical epidemiology language is used, referring mainly to Elisabeth Halloran book chapter [8] and to infectious epidemiology books [9–11]. The objective of this research is to present the trajectory of the epidemic over 3 years in Belgium. This paper is intended to help communication between actors in the field of infection control practice and data analysts.

## Material and methods

### Pragmatic definition of epidemic and epidemic waves

In common professional language, an “infectious epidemic” is characterised by a time trajectory of nonstationary variation in the number of new cases of infection in a cohort or in a population with three phases: increase, peak and decrease of incidence rate. No a priori standardised accepted mathematical criteria define infectious epidemic and infectious epidemic waves. For the COVID-19 propagation in the general Belgian population, the epidemic peaks were pragmatically defined *a posteriori* on two proposed criteria: 1°) peaks as maxima of the moving daily case reporting average rolling from 7 days before to 7 days after a specified date and 2°) to discard small fluctuations: the smoothed number of cases on peak day must be at least 30% above the number of cases two weeks before (peak day -14). When the interwave interval presented a period of fluctuations with multiple minimal values, the last prepeak minimal value and the first postpeak minimal value were taken as the starting date and ending date of successive waves. The interval period between two nonoverlapping epidemic waves – from the first postpeak minimal value of a wave to the last prepeak minimal value of the following wave – was defined as an interwave endemic period [12].

### From daily incidence to daily prevalence pool of infectious cases

The daily number of newly reported cases in Belgium were clinically defined during before April 2020 and confirmed by laboratory tests (practically, PCR Sars-Cov-2 tests) thereafter. Data were openly available through the databank of Sciensano, the Belgian public health institute [13]. This daily incidence rate of reported cases is symbolised as INCID. The same public open data bank shares the epidemic trajectories of VOC in a subsample of 5-10% of positive samples diagnosed in Belgium, obtained through sentinel laboratories (without clinical criteria of selection) considered representative of the whole country. VOC genomic sequencing began on 15 February 2021, i.e., when VOC Alpha was predominant.

Seroprevalence data after the first epidemic in cohorts representative of the general population have been published by another group [5], with their summary in Table 2. Seroprevalence after the second wave was taken from the Sciensano databank [13].

To be coherent with the SIR model, the daily prevalence pool I compartment represents the number of new COVID cases on a specified day INCID _day i_ + the number of prevalence pool of previous days still infected and infectious (prevalence pool I _day i-1_) [8] (the method of determining the prevalence pool I is developed in more detail in the Results section (Table 3)).

Ordinary differential equations of the SIR method were solved with a commercial computer software (Berkeley-Madonna Inc.) [14].This program fits the observed daily prevalence pool data (discrete data) to I of the SIR method (continuous function) by a recursive Runge‒Kutta method of numerical approximation [15]. The optimization algorithmic process of this software for obtention of parameters is based on the simplex method of Nelder-Mead for minimisation [16].

The statistical distribution of the epidemic parameters Ro, ß - infection rate or γ – recovery rate were not calculated with this computer software, and would require other programs based on Markov Chain Monte Carlo (MCMC) methodology (three chapters 9, 10, 11 on MCMC in reference [10]).

## Results

### Epidemiologic descriptive analysis of the three-year COVID-19 epidemic in Belgium

**Fig. 1:**
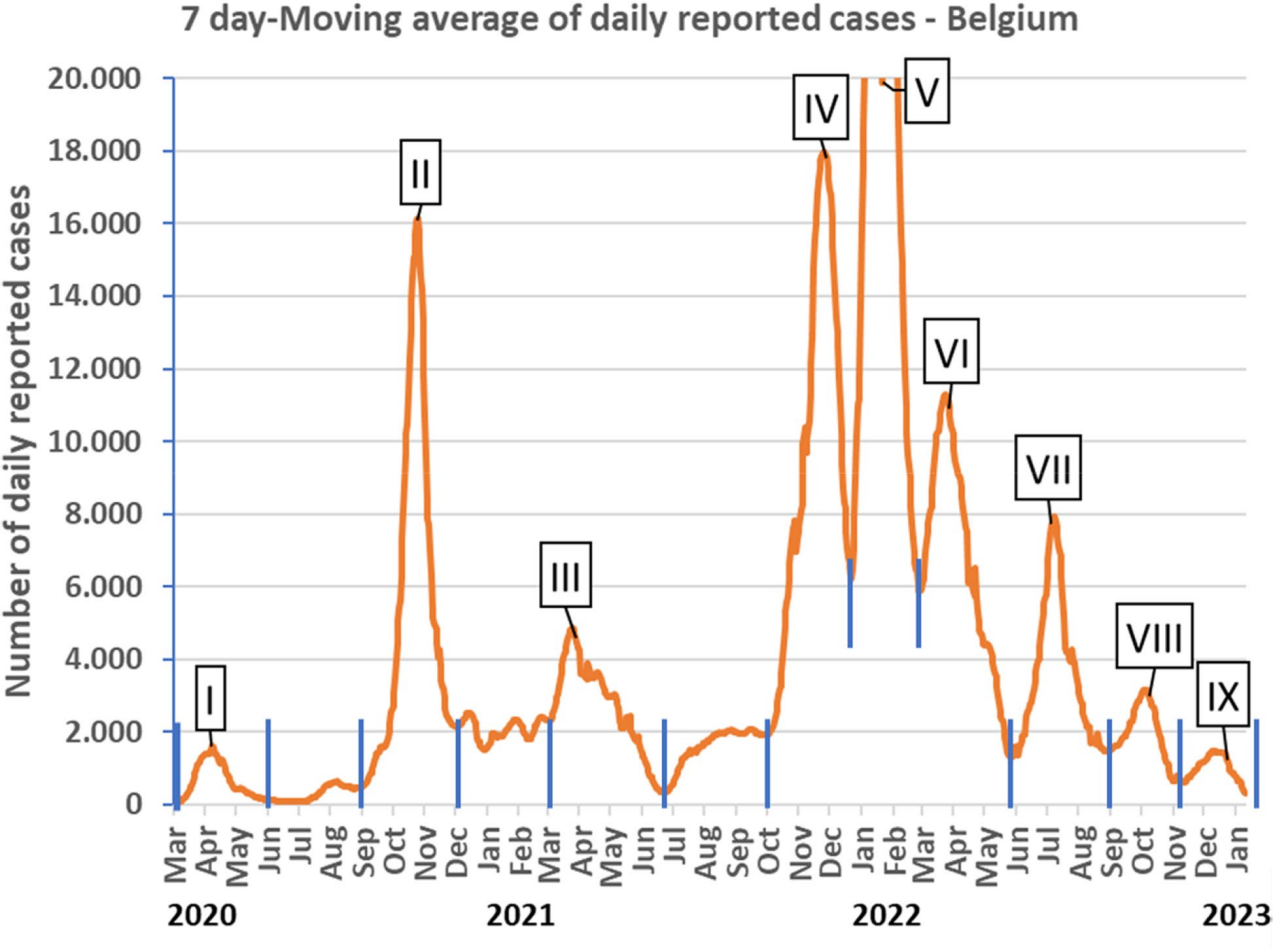
Descriptive analysis of the nine epidemic covid waves in general population – Belgium. Daily evolution of the number of Covid-19 reported cases smoothed with moving average from Day i-3 to Day i+3 (7 days moving average). Wave epidemic periods defined by peaks encircled by green vertical lines. Interwave endemic intervals defined by green vertical lines without interspersed peaks

When looking at the trajectory of reported COVID-19 incidence in Belgium (Fig. 1), visual inspection of the figure of smoothed daily reported case data shows a sequence of 9 waves numbered I to IX defined by their peaks and their prewave and postwave minimal values. These 9 waves were recognised over the three-year period March 2020 – begin January 2023. The first four waves were separated by three interwave intervals, I-II, II-III and III-IV, compatible with an unstable endemic interval. The last five waves overlapped without an interwave interval. The number of reported cases varied greatly, from < 2.000 cases-day^-1^ for wave I to 52.141 cases-day^-1^ for wave V (truncated at 20.000 cases-day^-1^ in Fig. 1).

Table 1 shows the descriptive analysis of the data after having defined the time milestones start - peak - end. The upper part describes the epidemic waves. The wave start times ranged over the four seasons: 4 in winter (waves I, III, V, VI), 1 in spring (wave VII), 2 in summer (waves II, VIII), and 2 in autumn (waves IV, IX). The length of the wave period varied from 66 days (Wave V) to 133 days (Wave III). Each wave was characterised by a predominant variant of concern. The number of reported cases at peak time and the total number during the wave reached a maximum during the fifth wave, at levels more than 20 times greater than during the first wave or the ninth wave. The lower part describes the three interwave periods/intervals for waves I to IV. The length of these intervals varied between 56 and 97 days. Due to fluctuating variations, there are no peaks clearly defined during these “endemic” intervals. The total number of cases during these intervals ranged from 20.848 during the first interwave period I-II to 160.138 cases for the third interwave period III-IV.

**Table 1:** Time milestones, duration, variant of concern, daily incidence no. at peak and wave amplitude of the nine Covid-19 epidemic waves - Belgium

**Epidemic wave (start year)**	**Start**	**Peak**	**End**	**Length** **(days)**	**Main** **WHO Variant VOC**	**No. reported cases**	
						**Peak**	**Wave**
Wave I (2020)	1 Mar	8 Apr	27 Jun	118	? Ancestral (Pre-VOC)	1.617	61.622
Wave II (2020)	24 Aug	25 Oct	1 Dec	99	? Wuhan (Pre-VOC)	16.147	503.678
Wave III (2021)	10 Feb	25 Mar	23 Jun	133	Alpha (B1.1.7)	4.857	346.034
Wave IV (2021)	30 Sep	25 Nov	22 Dec	83	Delta (B1.617.2)	17.982	796.193
Wave V (2021)	23 Dec	22 Jan	27 Feb	66	Omicron (BA.1)	104.540	1.529.867
Wave VI (2022)	28 Feb	23 Mar	6 May	87	Omicron BA.2 (BA.2)	11.292	385.454
Wave VII (2022)	28 May	10 Jul	3 Sept	98	Omicron BA.5 (BA.5)	7.915	338.848
Wave VIII (2022)	3 Sept	8 Oct	12 Nov	70	Omicron BA.F (BA.5)	3.167	134.735
Wave IX (2022)	12 Nov	16 Dec	> 9 Jan	119	Omicron BA.F (BA.5)	1.468	61.087
**Inter-wave period (start year)**	**Start**		**End**	**Length** **(days)**	**Main transition** **WHO Variant VOC**	**No. reported case Inter-Wave**	
Inter I-II (2020)	28 Jun		23 Aug	56	? Ancestral (Pre-VOC) ➔ ? Wuhan (Pre-VOC)	20.848	
Inter II-III (2020)	2 Dec		4 Feb	62	? Wuhan (Pre-VOC) ➔ Alpha (B.1.1.7)	145.234	
Inter III-IV (2021)	24 Jun		29 Sept	97	Alpha (B.1.1.7) ➔ Delta (B.1.617.2)	160.138	

*WHO Variant VOC* World Health Organization Variant of Concern (Pango lineage). Viral genomic sequencing in Belgium became available during the Wave III (2021) epidemic. Previous viral genomes (? Ancestral and ? Wuhan) are inferred from European SARS-CoV-2 surveillance

The availability of SARS-CoV-2 PCR diagnostic tests was low until the end of 2020, and the magnitude of the first wave had to be compared to data obtained by seroprevalence data reflecting the cumulative incidence of COVID-19, including clinical cases and asymptomatic cases [5, 17]. Table 2 shows that during the 20 April to 13 June 2020 period, i.e., the *a posteriori* cumulative incidence of Wave I, the seroprevalence of SARS-CoV-2 antibody-positive tests varied between 4,74% and 5,25%. Extrapolating to the general 11.4 million Belgian population, this represents an approximate number of 540 thousand cases of seroimmune conversions during the first wave, to be compared to the 9 times lower number of 61.622 reported cases by PCR during the first wave in Table 1.

**Table 2:** Post-Wave I SARS-CoV-2 seroprevalence data on a large sample collection in general population through outpatients referred to medical lab’s in Belgium in 2020 (reference [5]) and post-Wave II SARS-CoV-2 seroprevalence in blood donors (reference [13]). Last column: estimated no. of cases from seroprevalence data on basis of a 11,4 million Belgian population

**Period collection**	**Median seropositive prevalence (CI95) after wave I**	**Estimated total no. seroconversions during Wave I**
20 to 26 Apr 2020 18 to 25 May 2020 8 to 13 June 2020	5,25% (4,22% - 6,35%) 6,20% (5,05% - 7,31%) 4,74% (3,65% - 5,85%)	$\frac{\mathrm{5,25}\%+\mathrm{6,20}\%+\mathrm{4,74}\%}{3}$*11,4 millions ≈ 540 thousand seroconversions
		**Seroconversions during Wave II**
First week 2021	18,70% (15,60% - 21,70%)	(18,70%*11,4 millions) – 540 thousand ≈ 1,6 million seroconversions

Seroprevalence after wave II was available in blood donors, showing that 18,70% of this cohort was infected by SARS-CoV-2 during the first two epidemic waves. When subtracting the estimated number infected during the first wave in the general population, an estimated 1.6 million seroconversions during the second wave is calculated. This number is three times greater than the number of reported cases during the second wave in Table 1.

Table 3 shows the way to convert prevalence pool I from the daily incidence rate INCID. The pooled prevalence I corresponds to the sum of the daily incidence rate + the number of subjects having been infected previously and still infected and infectious. When a cohort is defined by its initial incidence rate **INCID**_**i**_, the number of subjects of this cohort remaining infected and infectious on the following days decreases as a theoretical exponential function with a coefficient rate γ:

**Table 3:** Conversion of daily incidence rates INCID _day i_ to daily prevalence pool I _day i_ during follow-up of an epidemic. Initial values of reported daily incidence rates are in bold characters. Each column represents the expected daily number of cases remaining infected and infectious during follow-up according to the exponential decrease of *INCID initial* with exponential decrease rate (γ coefficient). The prevalence pool I _day i_ (last column) corresponds to the sum within each row. The calculus of the prevalence pool is simplified by summing the incidence of the day i and the prevalence pool of the preceding day multiplied by number e exponent -γ: $Prevalence\;pool\;I_{\;day\;i}\;=\boldsymbol I\boldsymbol N\boldsymbol C\boldsymbol I{\boldsymbol D}_{\boldsymbol d\boldsymbol a\boldsymbol y\boldsymbol\;\boldsymbol i}\;+\;Prevalence\;pool\;I_{\;day\;i-1}\ast\;e^{-\gamma}$

**Day i**	$\mathrm{\Delta\,INCIDi}=\mathrm{initial}\,{\mathbf{I}\mathbf{N}\mathbf{C}\mathbf{I}\mathbf{D}}_{\mathbf{d}\mathbf{a}\mathbf{y}\mathbf{i}}\,\mathbf{*}{{\text{e}}}^{-\upgamma\,*(\mathrm{day\,i}-\mathrm{initial\,day\,i})}={\mathrm{\Delta\,Prevalence\,pool}}_{\mathrm{\,day\,i}-1}\mathbf{*}{{\text{e}}}^{-\upgamma\,}$						Preval. pool I _day i_
0	**INCID**_**0**_						I_0_=INCID_0_
1	INCID_0_*e^-γ*1^	**INCID**_**1**_					I_1_=INCID_1_ + I_0_*e^-γ^
2	INCID_0_*e^-γ*2^	INCID_1_*e^-γ*1^	**INCID**_**2**_				I_2_=INCID_2_ + I_1_*e^-γ^
3	INCID_0_*e^-γ*3^	INCID_1_*e^-γ*2^	INCID_2_*e^-γ*1^	**INCID**_**3**_			I_3_=INCID_3_ + I_2_*e^-γ^
4	INCID_0_*e^-γ*4^	INCID_1_*e^-γ*3^	INCID_2_*e^-γ*2^	INCID_3_*e^-γ*1^	**INCID**_**4**_		I_4_=INCID_4_ + I_3_*e^-γ^
5	INCID_0_*e^-γ*5^	INCID_1_*e^-γ*4^	INCID_2_*e^-γ*3^	INCID_3_*e^-γ*2^	INCID_4_*e^-γ*1^	**INCID**_**5**_	I_5_=INCID_5_ + I_4_*e^-γ^

1 $$\mathrm{\Delta\,INCID\,i}=\mathrm{initial\,}\,{\mathbf{I}\mathbf{N}\mathbf{C}\mathbf{I}\mathbf{D}}_{\mathbf{d}\mathbf{a}\mathbf{y}\mathbf\,{i}}\,\mathbf{*}{{\text{e}}}^{-\upgamma\,*(\mathrm{day\,i}-\mathrm{initial\,day\,i})}$$

(vertical columns in Table 3). The exponential coefficient γ corresponds to the transition coefficient I to R in the SIR model. The prevalence pool I_i_ (last column in Table 3) is the sum on day i of the values obtained in a row. The equation can be greatly simplified by observing that prevalence pool I follows the structure of a series with two terms [18]: the incidence rate of the day and the prevalence pool of the previous day multiplied by e^-γ^:

2 $$\mathrm{Prevalence\,pool\,}\,{{\text{I}}}_{\mathrm{\,day\,i}}={{\text{INCID}}}_{\mathrm{day\,i}}+\mathrm{Prevalence\,pool\,}\,{{\text{I}}}_{\mathrm{\,day\,i}-1}*{{\text{e}}}^{-\gamma}$$

**Fig. 2:**
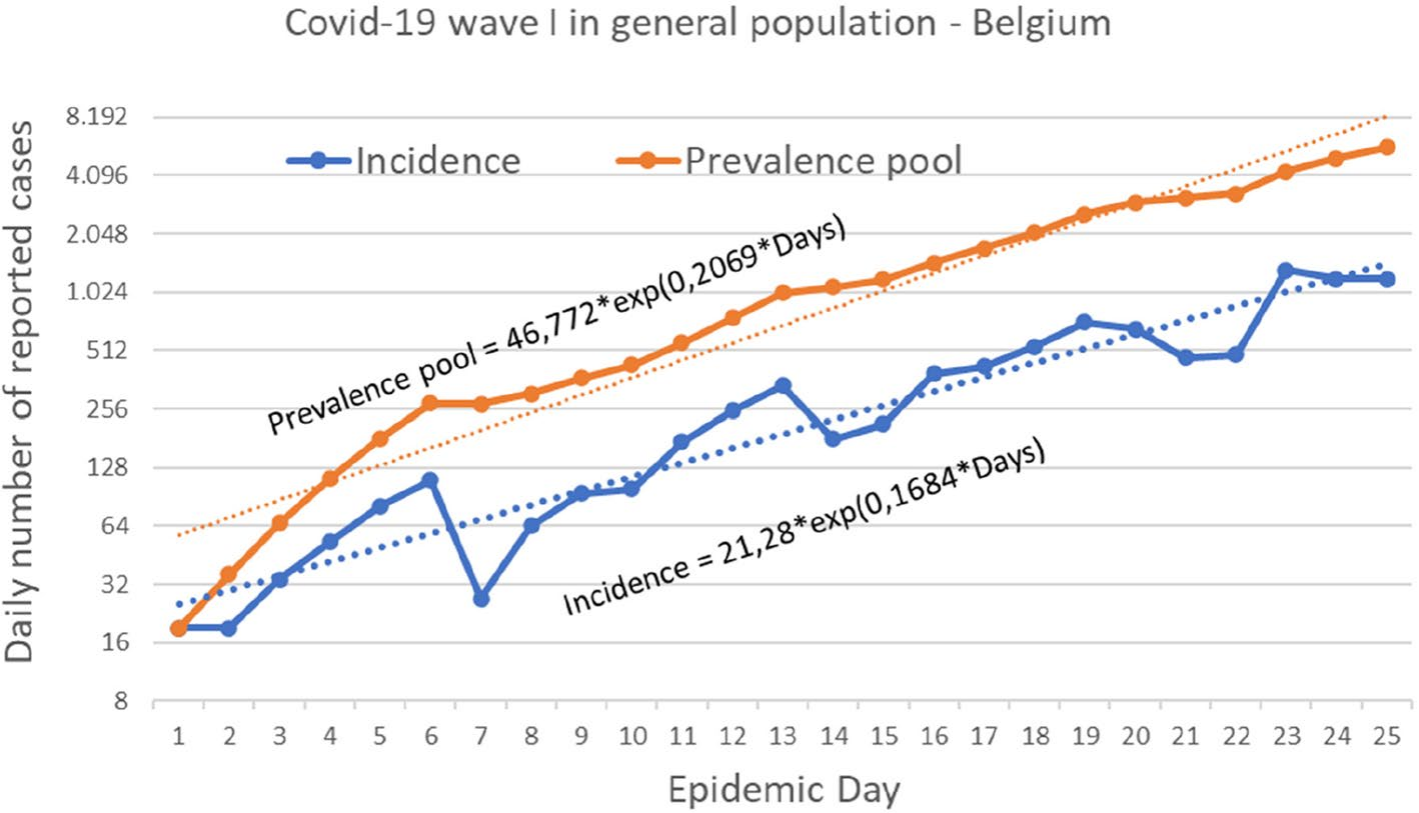
Comparison of prevalence pool I and incidence rate during first wave. Daily number of reported cases during the first 25 days of the first Covid-19 wave in Belgium, from 1st to 25 March 2020. Incidence = daily incidence rate. Prevalence pool calculated according to equation in Table 3: prevalence pool _(day i)_ = INCIDi + prevalence pool _(day i-1)_ * e^-γ^

The early epidemic phase during the first 25 days of COVID-19 epidemic wave I (between 1 and 25 March 2020) is analysed as an exponential function model (Fig. 2). Curves are compared for y-values expressed as daily incidence of reported cases (INCID) or as calculated prevalence pool I. The growth rate coefficient r value was 0,1684 day^-1^ for INCID and 0,2069 day^-1^ for Prevalence Pool.

### Determination of the basic reproductive number R_0_

Determination of the basic reproductive number *R*_*0*_ necessitates the knowledge of growth coefficient *r* = 0,2069 day^-1^ for prevalence pool I (Fig. 2) and the knowledge of serial time interval defined as the time interval between the onset of symptoms in the primary (infector) and secondary case (infected). Pragmatically, a close approximation of serial time is obtained by the generation time *Tg* defined as the sum of the average latent period (from contamination to infectiousness) and half the average infectious period. The generation time *Tg* reported from a meta-analysis in the clinical literature for SARS-CoV-2 corresponds to a mean incubation time of approximately 5 days [19] + half the mean duration of viable shedding of virus in the general population of approximately 8.4 days [20]. Estimated *Tg* = 9,2 days will be chosen as the initial value at this step for all epidemic waves and will be fitted later by analysis of the SIR model:

3 $$\mathrm Tg\;=\;\mathrm{mean}\;\mathrm{incubation}\;\mathrm{time}\;+\;\frac12\;\mathrm{mean}\;\mathrm{duration}\;\mathrm{of}\;\mathrm{viable}\;\mathrm{viral}\;\mathrm{shedding}=5\,\,\mathrm d\mathrm a\mathrm y\mathrm s+4,2\,\mathrm d\mathrm a\mathrm y\mathrm s=9,2\,\mathrm d\mathrm a\mathrm y\mathrm s$$

The inverse of time generation 1/*Tg also* corresponds to γ, the coefficient of transition of I➔R in the SIR model:

4 $$\gamma\,=\frac{1}{Tg}=\frac{1}{\mathrm{9,2}\,days}=\mathrm{0,11}\,{{\text{days}}}^{-1}$$

The basic reproductive number *R*_*0*_ was estimated on the basis of the growth rate r of the prevalence pool and the *Tg* generation time fixed at 9.2 days from the medical literature [21, 22]:

5 $${R}_{0}\,=\,\left({\text{r}}*\mathrm{Tg\,}+\,1\right)\approx\,\left(\mathrm{0,2069\,}*\mathrm{\,9,2}\right)+\,1\,=\mathrm{\,2,9035}$$

When having determined R_0_ and γ, the transition coefficient β, “force of infection”, between compartments S to I in the SIR model is calculated as follows [9, 21]:

6 $$\upbeta = R0^{\ \ast}\ \gamma = \text{2,9035}^{\ \ast}\ \text{0,11 day}^{-1} = \text{0,3194 day}^{-1}$$

### Fitting the observed prevalence pool I data of wave 1 to open the SIR model

**Fig. 3:**
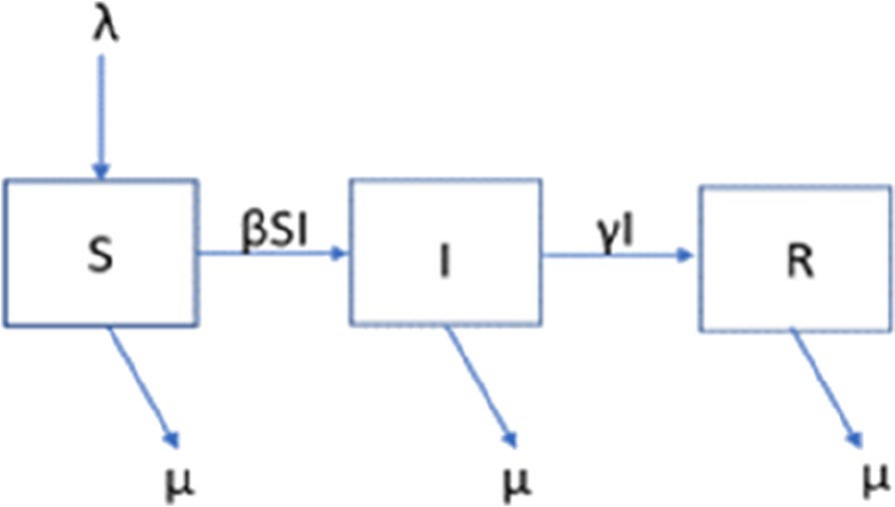
Open SIR model of epidemic trajectory. Dynamic model of evolution of an epidemic within an open cohort or population (i.e. λ as entry of new susceptible cases by births, immigration or in-travels being equal to µ (deaths)). S = Susceptible compartment, I = Infectious compartment, R = Recovered compartment. βSI = product of transition coefficient ß by S compartment and by prevalence pool I. γI = product of transition coefficient γ by prevalence pool I

The boxes and arrows (Fig. 3) represent the sequence of compartment Susceptible ➔ Infectious ➔ Recovered, with the transition coefficients as Greek letters β, γ for S to I and I to R transitions. To maintain a stable N number, λ as a parameter of entry in the S compartment is in equilibrium with µ representing mortality, migration or travel for exit of the S, I and R compartments. The “open” SIR model implies exchange inside a population by demographic movements with other groups.

To fit the prevalence pool I data to the SIR model, initial values of S, I and R have to be fixed.

Initial S value (S__time_0_): for an epidemic with a new pathogen, initial susceptible population S__time_0_ corresponds to N = the cumulative incidence of infected subjects during an epidemic wave plus the initially susceptible subjects who have escaped to the epidemic wave symbolised as S__time_∞_. The value of S__time_∞_ is obtained by Equation 7 (N, S__time_0_ and S__time_∞_ expressed in percentages). By the bisection method [23], a value of S__time_ ∞_ = 6,15% is obtained, with N = S__time_0_ = 100% for a new epidemic and R_0_ = 2.9035:

7 $$\begin{aligned}R_{0} &= \frac{Ln({S\_time\_0/S\_time\_}\infty)}{N-{S\_time\_}\infty} = \text{2,9035}\\ &\rightarrow {\text{S}\_\text{time}\_}\infty \end{aligned}$$

To determine the estimated N number of cases exposed to epidemic wave 1, the observed cumulative number of reported infections during the epidemic wave (61.622 cases) is divided by (100% - S__time_∞_):

8 $${\text{N}}=61.622/\left(100\%-\mathrm{6,15}\%\right)= 65.703 \approx 66.000$$

Initial value I was initially fixed as the number of initial prevalence pools in Fig. 2 (47 cases). It was modified to 200 cases to better fit the SIR model to the observed data.

Initial value R (R__time_0_) – recovered pool – was initially fixed as null for a new emerging virus.

**Fig. 4:**
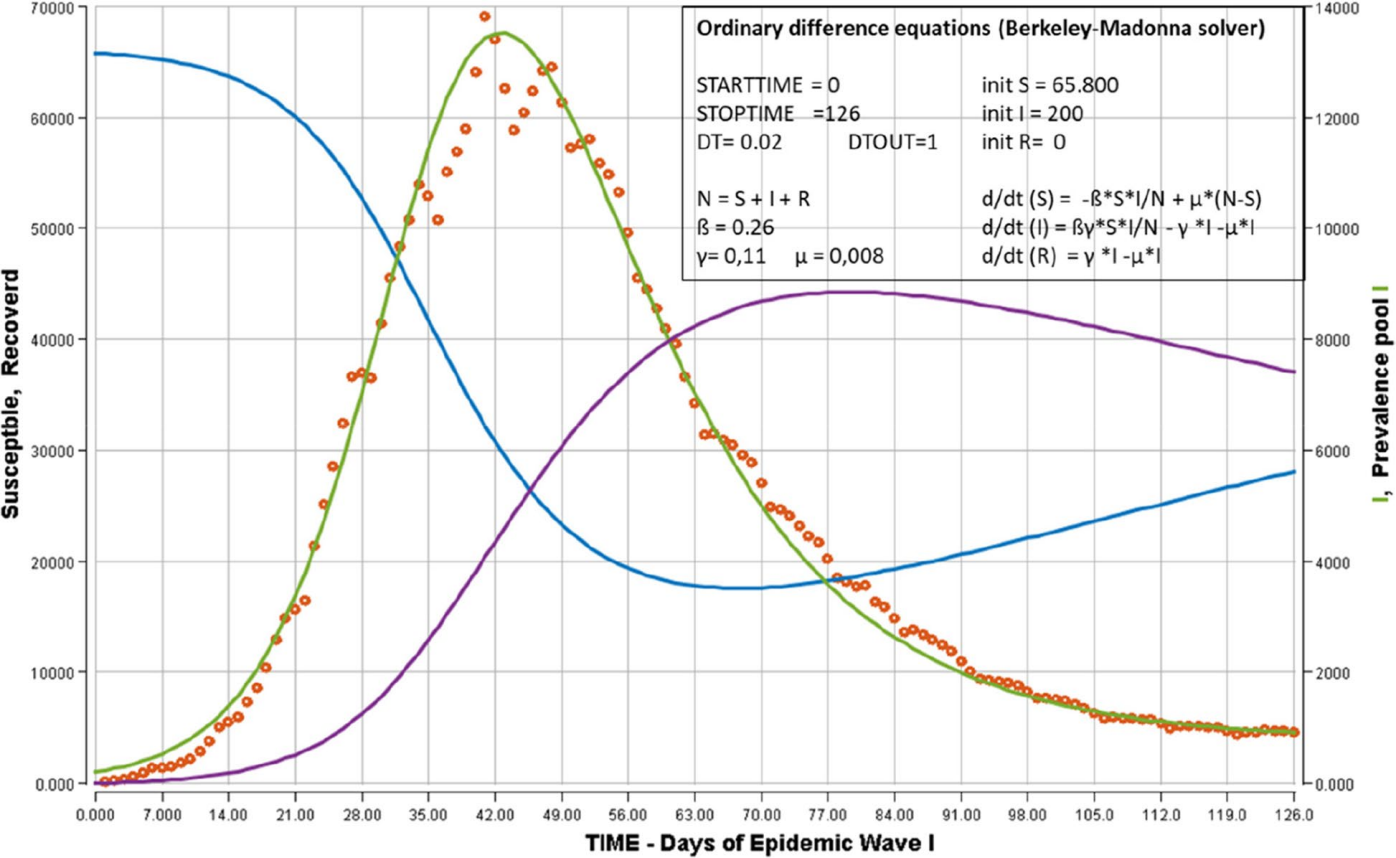
Open SIR model fitted to observations of the first Covid-19 epidemic wave – Belgium. The evolution of daily prevalence pool I was estimated from the daily incidence of reported cases (red circles). These observed values were fitted by ordinary differential equations (ODE) to SIR model. Left vertical axis: modelized Susceptible compartment (blue); modelized Recovered compartment (violet). Right axis: modelized daily prevalence pool I compartment. The insert contains the equations of ODE in Berkeley-Madonna language, the initial values of S, I and R compartments, the ß and γ transition parameters. DT signifies that the fitting of model to observations with ODE was operated for each Δ time = 0,02 days. DTOUT indicates the Δ time for output printing

Figure 4 shows the trajectory of prevalence pool I(t) and of the other compartments S(t) and R(t) after fitting the observed data of the prevalence pool by ordinary difference equations. The initial values of the number of cases in each compartment were as follows: N = 66.000 ≈ S__time_0_; I__time_0_ = 200; R__time_0_ = 0. The initial attributed values of the transition coefficients were as follows: ß for S ➔ I = 0,3194 (Equation 6); γ for I ➔ R = 0,11 (Equation 5). Initial I__time_ 0_ value and transition coefficients β and γ were submitted to fitting, with a domain of fitting between 0 and 500 for I__time_0_, between 0.15 and 0.45 for β, and between 0.08 and 0,20 for γ. After fitting the ordinary difference equations with Berkeley-Madonna software based on Nelder-Mead algoritms [16], the adjusted values of β and γ were, respectively, 0.39306 and 0.13083 (to be compared to their initial values of, respectively, β = 0,3194 (equation 4) and γ = 0.11 in equation 2). The initial value of I I__time_0_ was fixed at 200 (to be compared to the initial value of I__time_0_ = 47 cases obtained from Fig. 2). The prevalence pool I trajectory (green line) shows that the modelized curve was close to the observed data (open red circles) over the entire period. The susceptible compartment (blue, left axis) decreased from a maximal initial value of 66.000 in a classical sigmoid shape and attained a final minimal value S__time_∞_ = 786 at day 126 (representing 1,19% of S__time_0_ = 66.000), a much lower proportion than the expected S__time_∞_ = 6,15% of S__time_0_ in equation 6. The recovered compartment (violet line, right axis) followed a shape symmetrical to that of the susceptible compartment.

### Open SIR model (with demographic movements): expectation after first wave

**Fig. 5:**
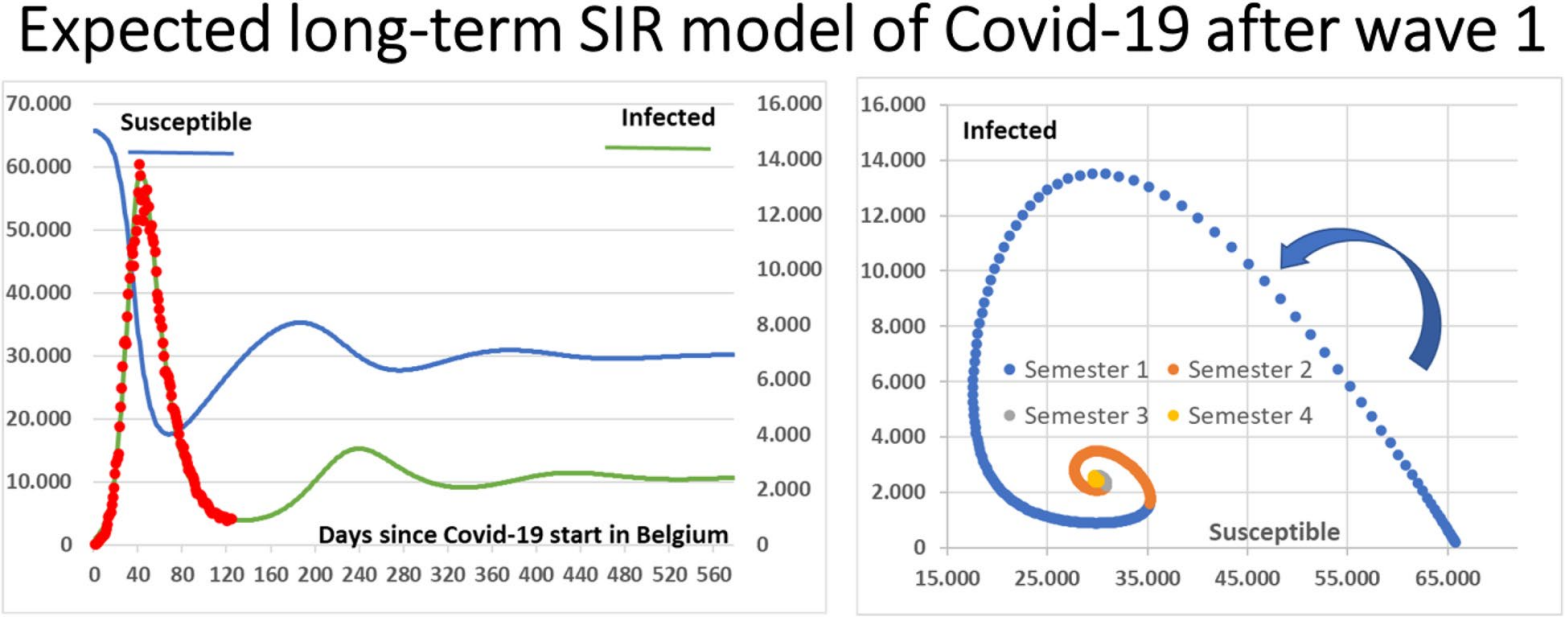
Expected recurrency by open SIR model after first wave of Covid-19 in Belgium. Left part: red circles: reported prevalence pool I data for wave 1 epidemic. Lines: prevalence pool I (green line, right axis) and Susceptible compartment (blue line, left axis) fitted to open SIR model for epidemic wave 1 with time extension to 580 days. Same initial S, I, R values and ß, γ transition values as in Fig. 4. Right part: the Susceptible – prevalence pool I plane representation shows that the model predicts a semestrial period of recurrence (the semestrial period being represented with different colors, initial period in blue and time direction indicated by the blue arrow) through a progressively stable endemic state after 4 semesters

Classically, an open SIR model evolves as a recurrent emergence of new waves with the same pathogen until an endemic stable plateau is reached. When the cohort to new arrivals (births, travellers) reaches an equilibrium between new infections and new entries, a stable endemic state is attained progressively. Figure 5 describes such a process. The same values for initial variables S__tim_0_ , I__time_0,_ R__time_0_ and for transitions coefficients ß, γ were employed as in Fig. 4, and the horizontal time axis was extended from 126 days (Fig. 4) to 600 days (Fig. 5). According to the model, the left part of Fig. 5 shows that when extending the model from the first epidemic wave to a period of 600 days, epidemic recurrence is expected with a semestrial periodic duration and progresses toward a stable endemic state of equilibrium. According to the SIR model, a significant proportion of the S__time_0_ cohort remains infected at the end of an epidemic wave and guarantees a new dampened epidemic wave when new susceptible subjects are introduced – or when public health measures are less strict. To keep N stable, the rate of introduction of new susceptible subjects symbolised by λ is artificially forced to be equal to μ, the rate of exit of compartments S, I and R.

### The emergence of new variants of concern

**Fig. 6:**
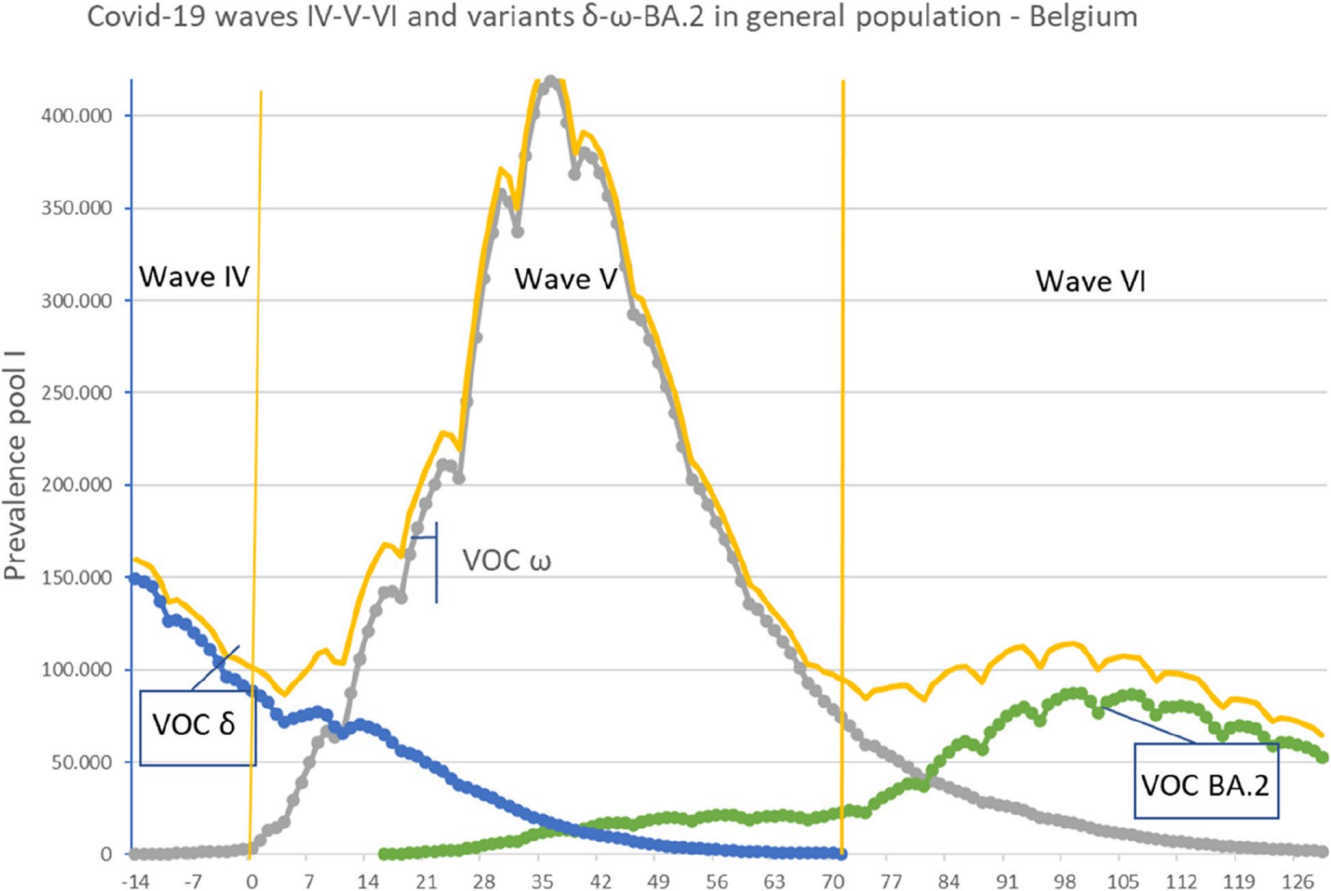
Trajectories of VOC SARS-CovV2 variants during epidemic waves IV, V and VI. Prevalence pool I (yellow line without marks) subdivided according to VOC Sars-CoV-2 variable of concern Delta (blue circles, VOC δ), Omicron (grey circles, VOC ω) and Omicron BA.2 (green circles, VOC BA.2)

To better understand the interplay between variants of concern VOC and epidemic waves of reported cases (Fig. 6), the epidemic trajectories of waves IV (VOC Delta queue of the trajectory), V (Omicron) and VI (VOC Omicron subvariant BA.2 part of trajectory) are presented. When looking at the distribution of variants of concern, before the initial day of wave V, there is a progressive decrease in the VOC Delta of the preceding wave and a progressive increase in VOC Omicron. At the end of this wave (after Day 70), there is a progressive decrease in VOC Omicron and a progressive increase in Omicron subvariant BA. The total number of reported cases (yellow line) corresponds to the summing of these VOCs.

**Fig. 7:**
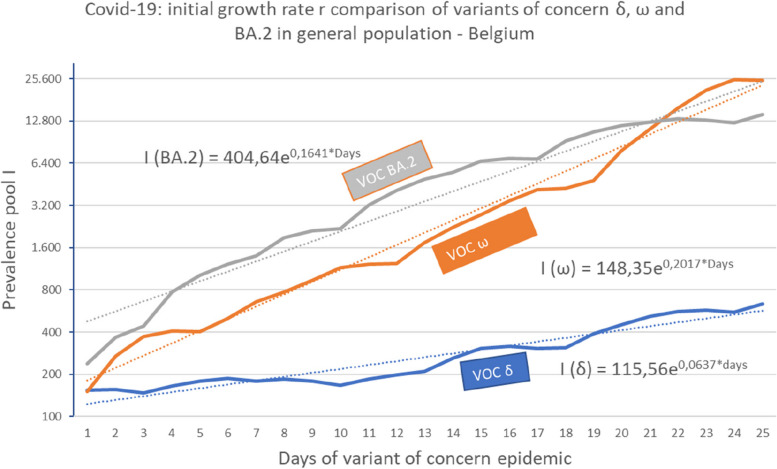
Comparison of growth rates of three Sars-CoV-2 variants of concern. Exponential growth during the 25 first days of variant wave epidemic. Right vertical axis: logarithmic scale on basis 2 for prevalence pool I. The exponential functions *I*(*VOC*) = *I*_*_time*_0_ * *e*^r*days^ are fitted to the observed data with I__time_0_ as initial value and r as growth rate for each of the three variables of concerns (δ, ω and ω subvariant BA.2)

To further analyse the exponential phase, each VOC is analysed individually. Such data covering the early phase of propagation of a new VOC have only been available since VOC Delta emergence in Belgium [13] (VOC Alpha sequencing began to be registered too late after its emergence). Figure 7 compares the growth rates of three VOC deltas, Omicron and Omicron BA.2. It is generally assumed that the amplitude of an epidemic wave is directly dependent on the growth rate, which is itself directly related to R_0_ by equation 5. Nevertheless, even for VOC Omicron, which has the greatest epidemic amplitude in wave V, a small fraction of the general population was reported as having been affected (13,16%, i.e., 1.5 million of 11.4 million people). This discrepancy between elevated growth rate and limited propagation in a population is explained by public health measures (lockdown, personal equipment (masks), hand rubbing with antiseptic agents). Another evidence is the lack of persistence of transmission of a VOC variant from an epidemic wave to the following one in link with the emergence of a new VOC variant (see discussion).

**Fig. 8:**
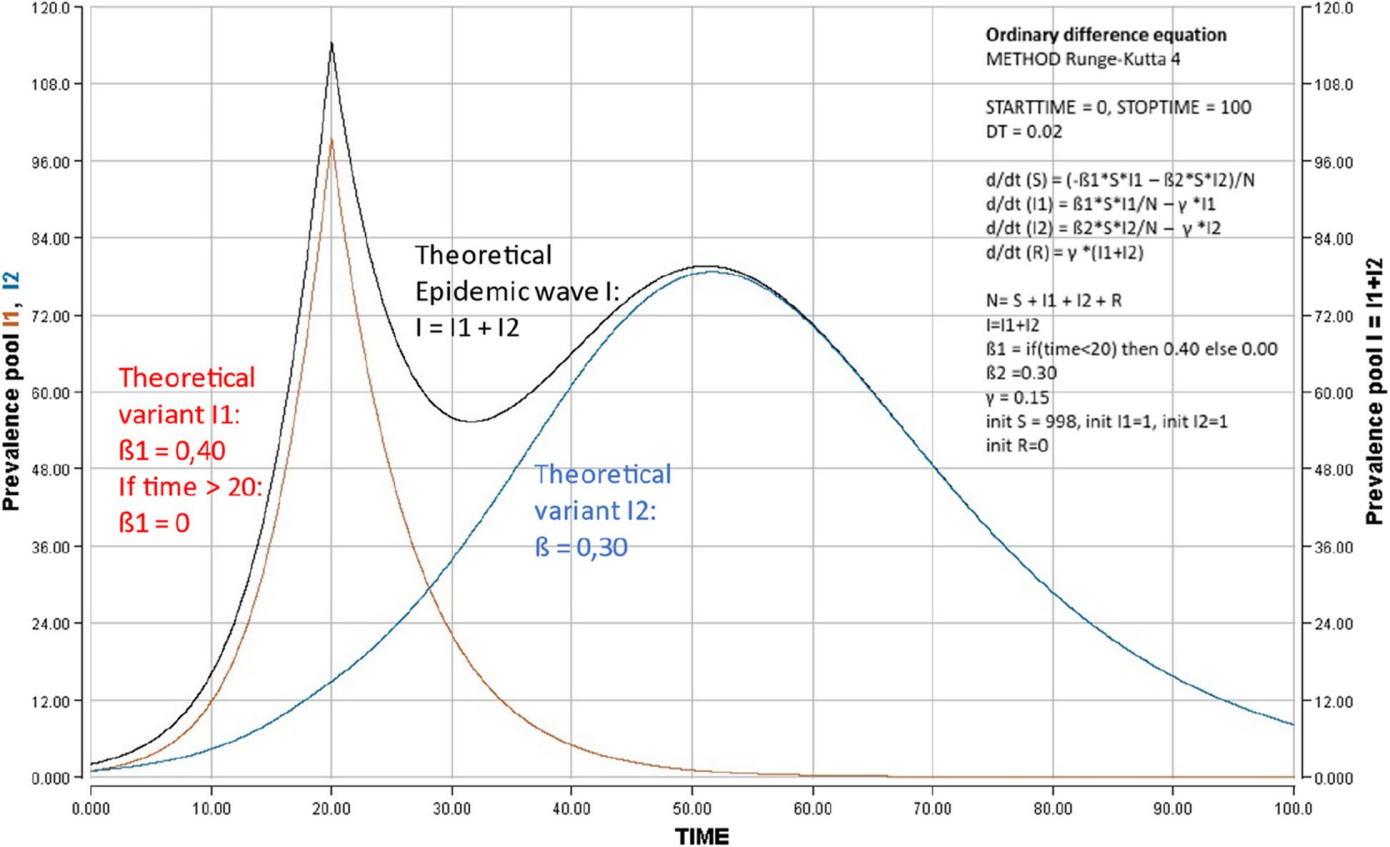
Forecasting an theoretical model of epidemic mixing two SARS-CoV-2 of differing transmissibility force. SIR model was analyzed by mixing two SARS-CoV-2 variants differing by their properties introduced simultaneously in a susceptible population at time 0. Variant 1 is more transmissible than variant 2 (transition coefficient S ➔ I ß1 for variant 1 = 0,40 versus ß2 for variant 2 = 0.30). But variant 1 has a relative short persistence (ß1 = 0 after 20 days) while variant 2 has an undetermined persistence (stable ß = 0.30 for more than 100 days). The graph shows that variant 1 has an initial advantage of propagation limited in time by its short persistence. the propagation of variant 2 with less transmissibility, but greater persistence in the population, predominates. Globally, variant 2 infects a larger proportion of the population. Insert: start-, stop- and delta (DT)-times, ordinary difference equations, initial compartment S, I, R values, transition coefficients S ➔ I ß, I ➔ R γ introduced for the analysis with Berkeley-Madonna software

Figure 8 visualises the model trajectory of a fictive epidemic due to two theoretical SARS-CoV-2 variants 1 and 2 with characteristics of propagation similar to those of SARS-CoV-2 (direct human to human transmission) emerging simultaneously in a population. Both variants differ by their initial ß transition coefficients (ß1 = 0.40 for variant 1 and ß2 = 0.30 for variant 2). Variant 1 has a limited number of cycles of transmission: after 20 days, ß1 equals zero. Variant 2 has no limited number of cycles of transmission (ß=0.30 remaining constant). In this model, the SIR model forecasts the coexistence of both variants, with a greater exponential growth of variant 1 versus variant 2 during the early phase. After the extinction of cycles of transmission of variant 1 on day 20, variant 1 is progressively replaced by variant 2. When analysing the total shape of both variants, variant 2 affects a markedly greater proportion of subjects than variant (75,1% by variant 2 versus 24,9% by variant 1) of initial total population (*N* = 1000).

## Discussion

The mathematical basis of a model of infectious epidemic was developed approximately one century ago, [1, 2] and its teaching continues to be largely employed in life sciences to understand the mode of pathogen epidemic transmission. In its simplest form of the SIR model, some assumptions are needed, such as “homogeneous mixing”, i.e., similar transmission in various categories of the population defined by age, sex, socioeconomic level, hospitalised/outpatient, etc. More powerful data analysis shows, for example, that SARS-CoV-2 transmission is an age-dependent variable [24]. Nevertheless, at an 11,4 million Belgian people population level, it may be considered that “homogeneous mixing” is sufficiently close to reality to infer approximately a simplified SIR model.

### Which data are correct at a population level?

The “representativeness” of collected clinical data is a major question of validity of the model: a nine-times greater number of cases during epidemic wave was reported a posteriori after wave I by seroprevalence (540 thousand cases) [5] versus the 61 thousand clinical reported cases to the national public health organism [13]. The same discrepancy between the total number of reported cases during wave II (504 thousand cases) and the number of cases determined after wave II from seroprevalence data (1,6 million cases) is documented. These discrepancies have partial explanations: a) *a posteriori* and before vaccination programs, approximately 40% of infected subjects escape a diagnosis (pauci- or asymptomatic cases) [25]; b) a lack of accessibility to PCR diagnostic tests at least during the 2020 year.

### Similar SIR pattern and transmission coefficients despite method-limited measurements

Nevertheless, even if the exact number of reported COVID-19 cases (including asymptomatic cases) remains underreported by daily incidence reporting, it may be assumed that the trajectory profile based on reported cases remains representative of its trajectory in the whole cohort of infectious cases measured *a posteriori* by seroprevalence before large vaccination programs. SARS-CoV-2 vaccination programs intended for the general adult population were introduced at the beginning of 2021: seroprevalence data were no more informative to determine the cumulative incidence of SARS-CoV-2 infections. At the end of 2021, 92% of the adult Belgian population > 18 years old had been vaccinated with at least one dose.

Despite these limits of methods of measurements, it is expected that the growth rate coefficient r, the transition coefficients ß (transmission rate) and γ (recovery rate) and the basic reproductive number R_0_ measured on basis of the reported cases adequately reflect the values of these parameters in the whole susceptible population, which is defined a posteriori as the total population having transited from S to R to I, with N being constant and even if the amplitude of a wave is underreported by clinical reporting. The SIR model bypasses this difficulty: the initial value of susceptible (S__time_0_ also symbolised by S_0_) is equal to the total number of subjects (generally symbolized by N), this number N remaining constant during the whole epidemic (N = S(t) + I(t) + R(t)). With this astute, the pattern of an epidemic SIR model remains identical whatever the number of cases observed – as long as this cohort is representative of a population, of course. In agreement with this observation, SIR model of the first wave trajectory based on daily clinical reporting of cases (Fig. 4, with S_0_ = 65.800 cases) or on a *a posteriori* cumulative incidence (Fig. S1, 658.000 cases) shows that the general profile of the epidemic trajectory is identical with identical transition coefficients β and γ and identical R_0_ values. None of these parameters measure the amplitude of an epidemic. This explains that during early phase of Covid-19 wave the predictions [26] were unconclusive on the expected amplitude of the epidemic.

The limit of propagation of COVID-19 in a population is also largely dependent on public health measures such as nonpharmaceutical interventions [27] and vaccination [28]. The principal limit of the measure of program efficacy is to refer to a comparative counterfactual group: how many people would have been infected in the absence of public health intervention? As shown above, this counterfactual number is largely dependent on the initially susceptible compartment number. It was generally assumed that the whole population was susceptible at the beginning of the pandemic with a new coronavirus. The limit of propagation of the virus to a small fraction of the population was attributed to the efficiency of public health programs. In this representation, a question arises when it is observed that a variant of an epidemic wave does not recur at the following wave. The question on SARS-CoV-2 persistence in the general population does not put in question the benefit of public health interventions; it is nevertheless essential to take it into account to measure the realistic efficacy and benefit of public health programs, particularly the expected amplitude of an emerging epidemic.

To date, it has been generally advocated that a progressive increase in transmission capacity explains the emergence and dissemination of new variants of concern, referring to the Darwin process of best survival of the fittest pathogen. When the basic reproductive number R_0_ of a new VOC is obviously greater than the R_0_ of a preceding wave, this representation is attractive. Nevertheless, when considering the coexistence of two viral strains with different basic reproductive numbers, the model predicts that both strains coexist in parallel, with greater propagation of the viral strain with greater R_0_. In other words, it is not expected that a more transmissible variant eliminates the less transmissible variant. An example of coexistence of viral strains is the case of yearly influenza epidemics: strains with different hemagglutinin A and B characteristics coexist jointly during the flu season [29]. The COVID-19 epidemic presents another trajectory: when a new variant emerges, the preceding variant disappears progressively.

### Open hypothesis for open discussion

An elevated rate of mutations [30] is described in the coronaviruses. Their genetic adaptation by mutation guarantees the relatively regular emergence of new variants of concern. Theoretically, “old” viral strains of VOC could be eliminated by defective mutations. Such a phenomenon is described *in vitro* in a large list of RNA viruses (Table 1 in [31]). Heterogeneity of viral fitness for replication (entry in the cell; replication and assembly within the cell and egress of the cell) is also described in some articles on SARS-CoV-2 [32]. Moreover, this speculative hypothesis could also give an alternative explanation to the observed progressive decrease of viral load during cross-sectional measurements of Ct values (Cycle threshold) of SARS-CoV-2 PCR measurements [33]. Note that in this paper, the authors associate this decrease of viral load by a modification of tested population (mainly tested in cases with acute phase of infection at the beginning of an epidemic wave and in cases with subacute or asymptomatic phase at the end of a wave).

With this concept in mind, it could explain the difficulty in predicting the true impact of a SARS-CoV-2 variant in a population: in addition to determining the transmission capacity of a variant by household transmission analysis [34, 35], stochastic model of early transmission [3] or S(E)IR transmission model [36], it would be necessary to predict the expected capacity of persistence of a VOC in a human population. In some severely immunodeficient patients, Sars-CoV-2 infection has been described as persisting for a long period, sometimes more than one year, with a same variant of concern [37]. In such cases, more than 30 mutations are registered per year and defective mutations (deletions in Spike gene domain) have been documented [38].

We did not try to put the variations of duration and amplitude of epidemic waves in relation to public health measures (lockdown and nonpharmaceutical interventions or vaccines) playing a major role in the transmission rate: our analysis of complex interplay between VOC was focused on a period of epidemic waves IV, V and VI when public health measures seemed grossly to be stable and strict in Belgium. Our analysis from the field opens a question of the variable persistence capacity of VOC in a population. Maybe cross-sectional studies not only of viral load [33], but also of virus fitness at various intervals during an epidemic wave could bring an answer to this up to now very speculative hypothesis.

## Conclusion

There is a need to clarify the methodological steps from the field data of COVID-19 epidemic characteristics to the use of these data in the SIR/SEIR model. The collection of data in the field requires clear definitions of the limits of methods in the representativeness of the sampling. The analysis of data into a mathematical model requires an effort of common wording between health workers and analysts. Evidence-based questions also arise from looking to observations: a limited persistence of SARS-CoV-2 variants of concern in the population and the absence of recurrence of a VOC at a following epidemic wave could be a source of variability in epidemic wave amplitude in successive VOC epidemics. The validity of this hypothesis is open to discussion.

### Supplementary Information

**Supplementary file 1.**

**Supplementary file 2.**

## Data Availability

The original datasets analysed during the current study are publicly available through the persistent web link https://epistat.sciensano.be/Data/COVID19BE_CASES_AGESEX.csv. The raw data analysis is shared through a supplementary file entitled “Sharing data analysis” associated with this manuscript.

## References

[1] Ross R (1916). An application of the theory of probabilities to the study of a priori pathometry Proceedings of the Royal Society of London (London)..

[2] Kermack WO, McKendrick AG (1927). A contribution to the mathematical theory of epidemics. Proceedings of the Royal Society of London (London)..

[3] Abrams S, Wambua J, Santermans E (2021). Modelling the early phase of the Belgian COVID-19 epidemic using a stochastic compartmental model and studying its implied future trajectories. Epidemics..

[4] Alleman TW, Vergeynst J, De Visscher L (2021). Assessing the effects of non-pharmaceutical interventions on SARS-CoV-2 transmission in Belgium by means of an extended SEIQRD model and public mobility data. Epidemics..

[5] Herzog SA, De Bie J, Abrams S (2022). Seroprevalence of IgG antibodies against SARS-CoV-2 – a serial prospective cross-sectional nationwide study of residual samples, Belgium, March to October 2020. Eurosurveillance.

[6] Grassly NC, Fraser C (2008). Mathematical models of infectious disease transmission. Nat Rev Microbiol..

[7] Rothman KJ, Greenland S. Prevalence. Prevalence, Incidence, and Mean Duration. In: Greenland S, Rothman KJ, editors. Modern Epidemiology. 3rd ed. Philadelphia, PA 19106 USA: Wolters Kluwer | Lippincott Williams & Wilkins; 2008. p. 42-4.

[8] Halloran ME. Concepts of infectious disease epidemiology. In: Rothman KJG, Sander, editor. Modern Epidemiology. Philadelphia, Pennsylvania: Lippincott-Raven Publishers; 1998. p. 529-54.

[9] Anderson RM, May RM (1991). Infectious diseases of humans.

[10] Held L, Hens N, O'Neil P, Wallinga J. Handbook of infectious disease data analysis. Fitzmaurice G, editor. New York: Chapman & Hall/CRC press; 2020.

[11] Brauer F, Castillo-Chavez C. Mathematical models in population biology and epidemiology. Marsden JES, L.; Golubitsky,M., editor. New York: Springer; 2001.

[12] Shaman J, Galanti M (2020). Will SARS-CoV-2 become endemic?. Science..

[13] Sciensano. Covid 29 datasets. 2023. https://epistat.sciensano.be/covid/. Accessed 9 Jan 2023.

[14] Marcoline F, Grabe M, Nayak S, Zahnley T, Oster G, Macey R. Berkeley Madonna User’s Guide Version 10.2.6 Berkeley Madonna, Inc., Albany, CA 94706. Berkeley Madonna,Inc., Albany, CA 94706. 2021.

[15] Hossain T, Miah M, Hossain B (2017). Numerical strucy of Kermack-Mckendrik SIR model to predict the outbreak of Ebola virus siseases using Euler and fourth order Runge-Kutta methods. Am Sci J Eng Technol Sci..

[16] Nelder JA, Mead R (1965). A simplex method for function minimization. Comp J..

[17] Cowling BJ, Wong JY. The use of seroprevalence data to estimate cumulative incidence of infection. In: Held L, Hens N, O'Neill P, Wallinga J, editors. Handbook of infectious disease data analysis. New York: Chapman & Hall/CRC Press; 2020.

[18] Marsden J, Weinstein A. 12.1. The sum of infinite series. In: Axler S, Gehring FW, Ribet KA, editors. Claculus II. 2nd ed. New York: Springer-Verlag; 1985. p. 561-70.

[19] Wu Y, Kang L, Guo Z, Liu J, Liu M, Liang W (2022). Incubation period of COVID-19 caused by unique SARS-CoV-2 strains: a systematic review and meta-analysis. J American Medical Assoc Netw Open..

[20] Cevik M, Tate M, Lloyd O, Maraolo AE, Schafers J, Ho A (2021). SARS-CoV-2, SARS-CoV, and MERS-CoV viral load dynamics, duration of viral shedding, and infectiousness: a systematic review and meta-analysis. Lancet Microbe..

[21] Maassen J. The SIR and SEIR Epidemiological Models Revisited. Preprints 2020. 10.20944/preprints202005.0090.v1.

[22] Anderson RM, May DH (1991). Infectious Diseases of Humans: Dynamics and Control.

[23] Marsden J, Weinstein A, Gehring FW, Halmos PR (1985). The method of bisection (Example 7). Calculus I.

[24] Franco N, Coletti P, Willem L (2022). Inferring age-specific differences in susceptibility to and infectiousness upon SARS-CoV-2 infection based on Belgian social contact data. PLoS Comput Biol..

[25] Oran DP, Topol EJ (2020). Prevalence of Asymptomatic SARS-CoV-2 Infection : A Narrative Review. Annals Int Med..

[26] Franco N (2021). COVID-19 Belgium: Extended SEIR-QD model with nursing homes and long-term scenarios-based forecasts. Epidemics..

[27] ECDC - European Center for Disease Prevention and Control. Guidelines for non-pharmaceutical interventions to reduce the impact of COVID-19 in the EU/EEA and the UK. . Stockholm. 2020. https://www.ecdc.europa.eu/en/publications-data/covid-19-guidelines-non-pharmaceutical-interventions. Accessed 15 Mar 2023.

[28] ECDC - European Center for Disease Prevention and Control. COVID-19 vaccination. Stckholm. 2023. https://www.ecdc.europa.eu/en/covid-19/prevention-and-control/vaccines. Accessed 15 Mar 2023.

[29] Han AX, de Jong SPJ, Russell CA (2023). Co-evolution of immunity and seasonal influenza viruses. Nat Rev Microbiol..

[30] Carabelli AM, Peacock TP, Thorne LG (2023). SARS-CoV-2 variant biology: immune escape, transmission and fitness. Nat Rev Microbiol..

[31] Vignuzzi M, López CB (2019). Defective viral genomes are key drivers of the virus–host interaction. Nature Microbiology..

[32] Jones JE, Le Sage V, Lakdawala SS (2021). Viral and host heterogeneity and their effects on the viral life cycle. Nature Reviews Microbiology..

[33] Hay JA, Kennedy-Shaffer L, Kanjilal S (2021). Estimating epidemiologic dynamics from cross-sectional viral load distributions. Science.

[34] Verberk JDM, de Hoog MLA, Westerhof I (2022). Transmission of SARS-CoV-2 within households: a remote prospective cohort study in European countries. Eur J Epidemiol..

[35] Harris RJ, Hall JA, Zaidi A, Andrews NJ, Dunbar JK, Dabrera G (2021). Effect of Vaccination on Household Transmission of SARS-CoV-2 in England. New England J Med..

[36] Mwalili S, Kimathi M, Ojiambo V, Gathungu D, Mbogo R (2020). SEIR model for COVID-19 dynamics incorporating the environment and social distancing. BMC Res Notes..

[37] Hettle D, Hutchings S, Muir P, Moran E, consortium C-GU. Persistent SARS-CoV-2 infection in immunocompromised patients facilitates rapid viral evolution: Retrospective cohort study and literature review. Clin Infect Pract. 2022;16:100210. 10.1016/j.clinpr.2022.100210. 10.1016/j.clinpr.2022.100210 PMC9666269 36405361

[38] Gandhi RT, Castle AC, de Oliveira T, et al. Case 40-2023: A 70-Year-old woman with cough and shortness of breath. New Engl J Med. 2023;389(26):2468–76. 10.1056/NEJMcpc2300910. 10.1056/NEJMcpc2300910 38157503

