# Supplementary figures and images for: Wording the trajectory of the three-year COVID-19 epidemic in a general population – Belgium
Source: BMC Public Health. 2024 Feb 29;24:638. doi: 10.1186/s12889-024-17951-x (PMC10903008; doi:10.1186/s12889-024-17951-x)

*Supplementary material*


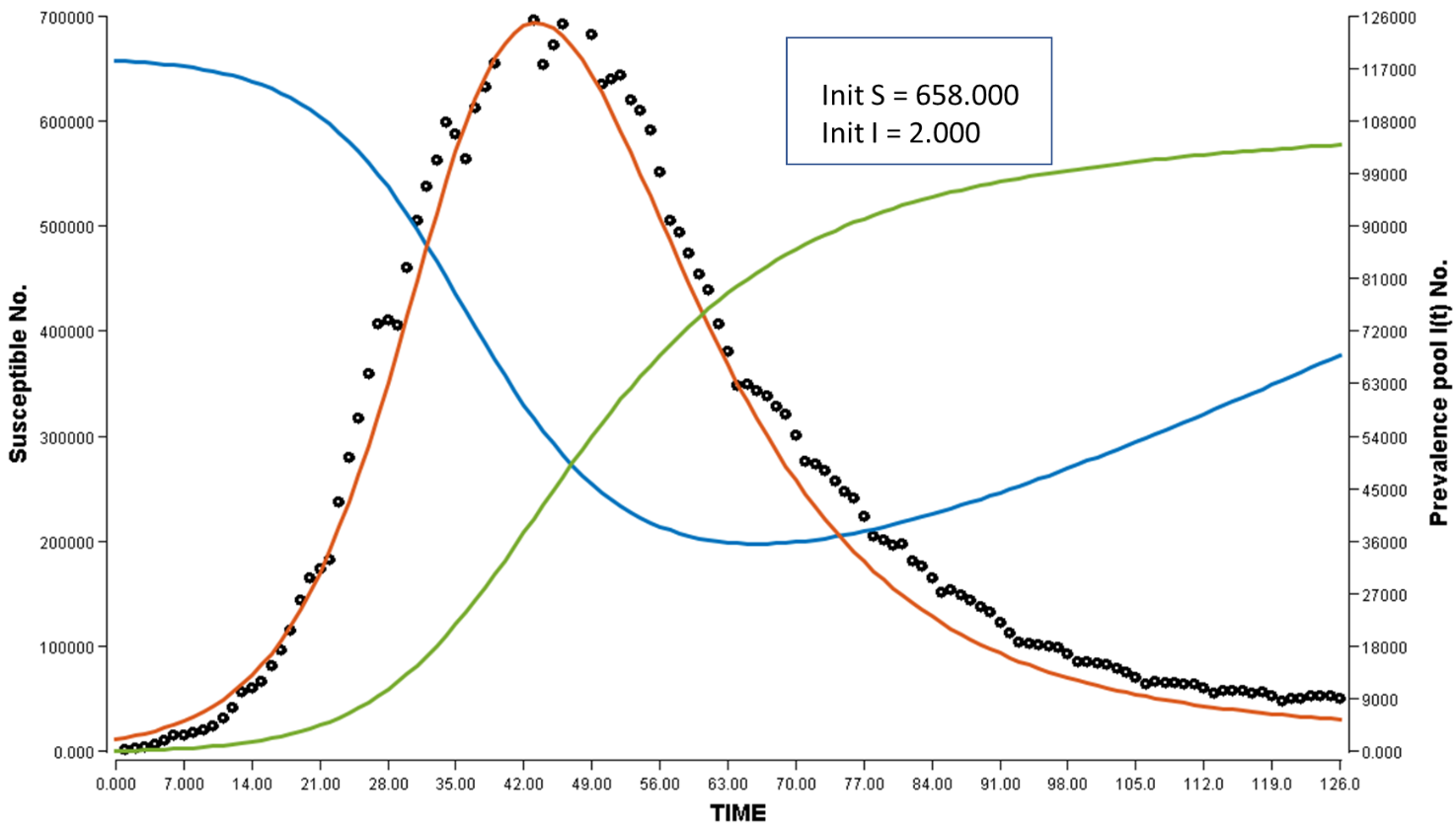


**Figure S 1.**

Supplement: Supplementary file 1 [file 12889_2024_17951_MOESM1_ESM.docx]
